# An Ending and a Beginning

**DOI:** 10.31486/toj.19.5010

**Published:** 2019

**Authors:** Ronald G. Amedee

**Affiliations:** Designated Institutional Official, Office of Graduate Medical Education, Division of Ochsner Academics, Ochsner Clinic Foundation, New Orleans, LA; Head of Clinical School and Professor, The University of Queensland Faculty of Medicine, Ochsner Clinical School, New Orleans, LA; Editor-in-Chief, *Ochsner Journal*

The Winter Solstice is the time of our ending and beginning, a powerful time…a time to contemplate your immortality. A time to forgive, to be forgiven, and to make a fresh start. A time to awaken.

–Frederick Lenz

This edition of the *Journal* includes another collection of strong and timely original research manuscripts, including an article by Simmons and Milburn entitled “Impact of Low-Dose Computed Tomography Orders and Scan Length.” Based on the belief that lower-dose CT scans pose less risk of radiation exposure to patients, Simmons and Milburn conducted a retrospective review to determine if the clinical application of adaptive statistical iterative reconstruction reduced the radiation dose in abdominal scans without changing ordering preferences, increasing the number of examinations requested, or increasing the CT scan coverage. A second article about radiologic imaging looks at the “Association of Computed Tomography With Treatment and Timing of Care in Adult Patients With Peritonsillar Abscess.” In their retrospective review, Carratola et al examined 3 outcomes—admission, bedside procedure (needle aspiration, incision/drainage), and surgical procedure (incision/drainage, tonsillectomy)—to determine if a diagnostic CT scan was associated with a difference in clinical intervention and with delaying the intervention.

Increasing numbers of medical school applicants have worked or are currently working as medical scribes, making “Medical Scribes in the Emergency Department: the Scribes’ Point of View” by Eley and Allen such an interesting read. Another important original research article to commend for your review is “Sexual Harassment in the House of Medicine and Correlations to Burnout: A Cross-Sectional Survey.” Mathews et al report sobering survey results about female physicians’ experiences with gender harassment and unwanted sexual attention. Yet another ripped-from-the-headlines article examines the staggering increase in fentanyl-related deaths in Jefferson Parish, LA from 2013 to 2018.

In addition to these fine papers, this issue contains 3 more important original research articles: “Complication Rates Following Septoplasty With Inferior Turbinate Reduction”; “Disease Spectrum and Frequency of Illness in Pediatric Emergency: A Retrospective Analysis from Karachi, Pakistan”; and “Incidence of Dysplasia in Obese vs Nonobese Patients With Nondysplastic Barret Esophagus.”

We have 3 robust contemporary updates in this issue: “Pediatric Attention-Deficit/Hyperactivity Disorder in Louisiana: Trends, Challenges, and Opportunities for Enhanced Quality Care” by Kumar and Gleason; “Cardiac Transplantation: Update on a Road Less Traveled” by Gupta and Krim; and “A Systematic Review of Interventions to Address Accent-Related Communication Problems in Healthcare” by Gu and Shah.

Under the heading Case Reports and Clinical Observations, we offer 4 interesting cases from the specialties of neurology, cardiology, sports medicine, and oncology: “A Rare and Treatable Cause of Medullar Claudication: Spinal Dural Arteriovenous Fistula” by Derollez et al; “Refractory Ventricular Tachycardia From Coronary Vasospasm During Pregnancy” by Ergle and Bernard; “Acute Traumatic Tear of the Gluteus Medius and Gluteus Minimums in a Marathon Runner” by Godshaw et al; and “Left Cervical Lymphadenopathy Presentation of Metastatic Colorectal Adenocarcinoma” by Maslov and Bragg.

This edition of the *Journal* is the final issue for 2019. I wish to thank all authors who have submitted their manuscripts to our publication for consideration and whose work has been published this year. We look forward to receiving more of your scholarly work in 2020. The *Journal* would not be possible without the expertise and dedication of our editorial staff who make each issue possible. For 2020, I wish each of our contributors and readers the “fresh start” Frederick Lenz refers to in his meditation on the solstice. Every new year brings us a powerful time of contemplation and the promise of new beginnings.

